# Pain as a Guide in Glasgow Coma Scale Status for Neurological Assessment

**DOI:** 10.21315/mjms2023.30.5.18

**Published:** 2023-10-30

**Authors:** Vasu Nallaluthan, Guan Yan Tan, Mohamed Fuad Murni, Umaira Saleh, Sanihah Abdul Halim, Zamzuri Idris, Abdul Rahman Izaini Ghani, Jafri Malin Abdullah

**Affiliations:** 1 Department of Neurosciences, School of Medical Sciences, Universiti Sains Malaysia, Kelantan, Malaysia; 2 Hospital Universiti Sains Malaysia, Universiti Sains Malaysia, Kelantan, Malaysia; 3 Department of Neurosurgery, Hospital Sungai Buloh, Selangor, Malaysia; 4 Department of Neurosurgery, Hospital Tengku Ampuan Afzan, Pahang, Malaysia; 5 Department of Neurosurgery, Hospital Sultanah Aminah, Johor, Malaysia; 6 Department of Neurosurgery, Hospital Pulau Pinang, Pulau Pinang, Malaysia; 7 Neurology Unit, Department of Internal Medicine, School of Medical Sciences, Universiti Sains Malaysia, Kelantan, Malaysia; 8 Brain and Behaviour Cluster, School of Medical Sciences, Universiti Sains Malaysia, Kelantan, Malaysia

**Keywords:** pain stimulus, GCS status assessment, supraorbital, jaw margin, sternal rub, periungual, pain pathway

## Abstract

Neurological status is essential and often challenging for neurosurgical residents and also for neurosurgeons to determine surgical management. Pain as a component of the Glasgow Coma Scale (GCS) can be used as a tool in patients, especially an unconscious or comatose patient. In order to elicit this adequate noxious stimulus, a certain amount of pressure-pain threshold is required upon performing either as the central or peripheral technique. The scientific explanation behind each technique is required and needs to be well understood to aid the localisation of the defect in the neurological system. This paper will briefly review the aid of pain as a neurological guide in GCS status assessment.

## Introduction

Subtle movement upon examination of the ranges of motor across limbs, face and ocular motility even in vegetative or unresponsive wakefulness state patients provides neuro-clinicians (1) a clue on neurological status especially via pain stimuli. Teasdale and Jennet (2) introduced a stereotyped clinical tool that any medical personnel is able to make rapid systematic evaluations of neurological status (3). Fairley and Cosgrove mentioned that applying adequate painful stimuli does not cause long-term pain or damage but enables an assessment of the level of consciousness (GCS) or the depth of coma in a patient who is in an altered state (3). Severity assessment in GCS will provide clinicians to decide whether or not severity is sufficient enough to justify certain types of treatment; to compare different series of injuries, particularly if alternative methods of management are on trial; and as a guide to the degree of ultimate recovery to be expected (4). The pattern of change in five physiological functions: i) state of consciousness; ii) breathing; iii) pupillary size and reaction; iv) eye movements, ocular reflexes and v) motor responses will provide a diagnosis of cause and site according to Plum and Posner (4). Response to pain in motor scale is a good basis for grading the depth of coma, especially in patients who cannot obey or speak (4). Painful or noxious stimuli are only applied to a patient who fails to open his/her eyes or respond to verbal voice commands spontaneously (3).

### Pain as 5th Vital Sign

The International Association for the Study of Pain (IASP) had define pain as an unpleasant sensory and emotional experience associated with actual or potential tissue damage or described in terms of such damage (5, 6, 21). Pain is the consequence of stimulation of pain-sensitive. When pain is the result of physiological activity in the normal pain receptors and there is no primary dysfunction of the nervous system, it is called nociceptive pain (5, 6). Nociceptive pain may indicate a disorder in any other system or organ, and its diagnosis and treatment involve different medical specialties (7). Any noxious stimuli must undergo three basic processes of pain mechanism which is transduction, transmission, and modulation (8, 9, 12). Upon experiencing pain, distributed processing involves the hypothalamus, internal capsule (lenticular nucleus), the anterior cingulate (ACC), insular cortex, thalamus, the somatosensory cortices, the superior and inferior frontal cortex, the straight gyrus and the cerebellar vermis (6). Mainly, there are two different types of painful or noxious stimuli: central and peripheral. Exerted pain on extremities such as nail bed (ungual) pressure technique is considered a peripheral stimulus (10). Central stimuli technique applied to the core of the body which consists of a trapezius muscle squeeze, supraorbital pressure, mandibular pressure or the commonly practiced sternal rub (2, 10).

### Neuroscientific Concept Behind Pain Stimuli

Perception of pain is initiated at the pain sensitivity or transduction stage, followed by signal transmission from the periphery to the dorsal horn (DH) (6) of the spinal cord via the peripheral nervous system (PNS). In brief, pain info from the skin of the head to the spinal and principal trigeminal nucleus, and from the neck mucosa to the lower 2/3rd of solitary tract nucleus (6). Subsequently to the brain, the higher level of CNS with the aid of ascending and descending pathways without fault (8). Meanwhile, prefrontal, M1, S2, anterior cingulate and insular cortices, the thalamus, supplementary motor areas, amygdala, PAG and basal ganglia plays important CNS components of the acute pain network (7).

Nociceptors that produce tissue damage due to stimuli response have free nerve endings of either A-δ a Type II (myelinated) or C fibres (unmyelinated) which are smaller-diameter primary afferents. The Aδ fibres allow the localisation of pain and create an afferent pathway for the reflexes elicited by pain stimuli (6, 11, 30). Internally nociceptors are found within muscles, joints, bones, internal organs, and externally in the skin with higher concentrations in the fingertips, hands, and face but lower concentrations over the torso (11, 12). Mechanical nociceptor afferents also present at skin, subcutaneous tissue and fascia. There are other specific receptors for different stimuli such as Piezo receptors (mechanical), TRP receptors (extreme temperature), P2X purinergic receptors (adenosine triphosphate [ATP]), ASICs (hydrogen ions), etc (9, 11) and they respond to chemical stimuli as well and are referred as polymodal nociceptors (11).

Upon pain pressure is applied, either nociceptor will get stimulated and transduce into neuronal action potentials. Transduction occurs along the nociceptive pathway. Initial stimulus events will be changed to chemical tissue events; then synaptic cleft events and subsequently changed into electrical events in the neurons, and electrical events in the neurons are transduced as chemical events at the synapses (8, 9, 30). Prostaglandins are produced when there is tissue damage (8, 11) which directly stimulates the nociceptors to accept the noxious stimulus. Potassium (K+), ATP and hydrogen ions also from the damaged tissue cells will also directly stimulate the nociceptors (9, 12).

The resting membrane potential (around −70 mV) downstream is raised until the threshold potential (around −55 mV) is achieved by stimulating the nociceptors’ open voltage-gated ion channels to allow calcium and sodium ions to pass into the cytoplasm, which subsequently leads to action potential formation (8, 12). The signal as an action potential travels up the primary afferent axon as it is propagated by the continued reaching of threshold potential due to the opening of voltage-gated Na^+^ channels upstream (saltatory conduction) (8, 12).

### Pain Stimulus Technique and Its Related Pathway

The pressure pain threshold is defined as the amount of pressure required to change a sensation of pressure to a sensation of pain (13). A mechanical pressure algometer is a device that is used to determine the pressure needed to convert the sensation of pressure to pain (13). The gauge will display values in kg/cm^2^ which means the amount of weight needed to convert the sensation of pressure to pain.

### Central Stimulus

#### Supraorbital Pressure Technique Pathway

**Figure 1:**
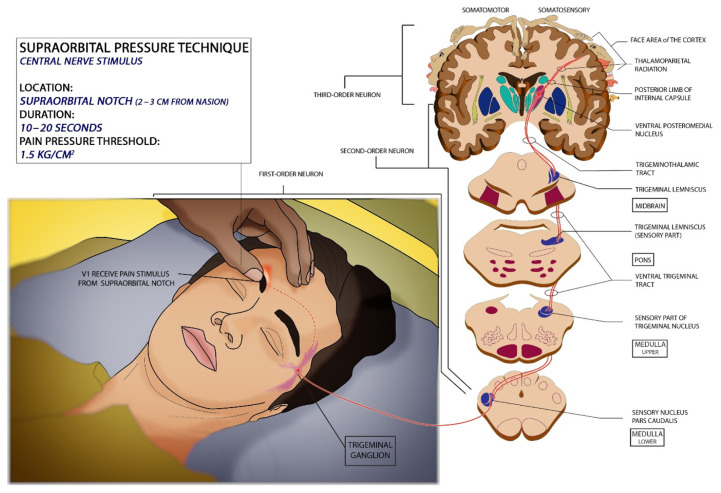
Illustration shows how to perform the supraorbital pressure technique and its prerequisite with related neurological pathways

A stimulus at the supraorbital notch (Figure 1) is applied primarily by identifying the orbital rim first and then locating the supraorbital notch (calculated by using a ruler measuring 2 cm–3 cm from nasion) (3, 9). Based on a study done on pressure pain threshold over the supraorbital nerve a mean pressure of 1.5 kg/cm^2^ is required to convert or elicit the sensation of pressure to pain or response from the patient (14, 15). Pain sensation stimuli will travel from the myelinated supraorbital nerve (branch of V1 of CN V) (3) and pass through the superior orbital fissure to reach the trigeminal ganglion in the Meckel cave (19). It then travels to the chief sensory nucleus located in the ventrolateral pons. It then ascends to the trigeminal lemniscus and travels to the ventroposterior medial (VPM) nucleus of the thalamus (19).

#### Jaw Margin Pressure Technique Pathway

**Figure 2:**
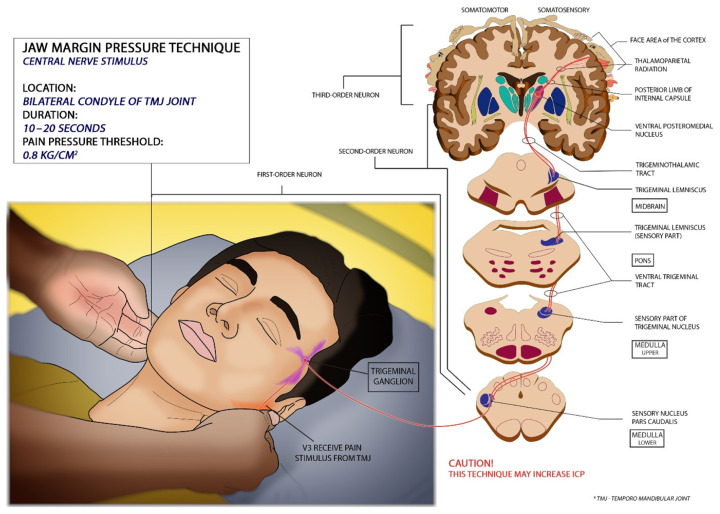
Illustration shows how to perform the jaw margin pressure technique and its prerequisite with related neurological pathways

Wijdicks jaw margin pressure (CN V) is an alternative method of applying central pain stimulus (15, 16). Application of deep pressure on both condyles at the level of the temporomandibular joint (Figure 2) is able to stimulate the pain (9, 15, 16). Pain is stimulated by placing the flat of the thumb on both condyles at the level of the temporomandibular joint (TMJ) and then applying pressure equivalent to 0.8 kg/cm^2^ (78 kPa) (17) with gradually increasing for a duration of 10 s–20 s to elicit a response (15). The technique needs to apply with caution in a patient with raised ICP, as this may increase further ICP if the venous return is compromised due to compression of the jugular vein. The pain impulse produced by the pressure on the temporomandibular joint capsule will be received by the auriculotemporal nerve of the third branch of cranial nerve V (16, 18). Additionally, a branch of the second branch of the trigeminal nerve; the masseteric nerve innervates the articular capsule in the anterolateral portion (19). The third branch of the trigeminal nerve provides the main nerve supply of the TMJ which passes through the trigeminal ganglion and synapse in the spinal tract (19). The spinal tract of the trigeminal nucleus synapse with second-order neurons, and crossovers to the contralateral side, forming the ventral trigeminal tract and ascending towards the VPM nucleus of the thalamus (19).

#### Sternal Rub Technique Pathway

**Figure 3:**
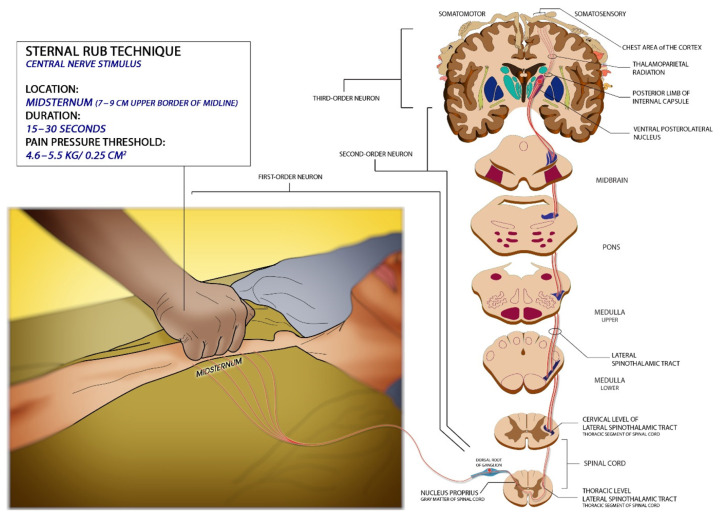
Illustration shows how to perform the sternal rub technique and its prerequisite with related neurological pathways

The sternal rub technique (Figure 3) is applied at the mid sternum (approximately between 7 cm and 9 cm below the upper border of the midline) (9). It is lack overlying muscle and is covered with little fat thus direct response to pressure stimuli can be achieved (19). Using the knuckles of the fist, rub vigorously in a *pestle and mortar manner* against the area for at least 15 s–30 s (9, 15, 20) before any response is seen. Pressure stimulation onto the sternum shall be ranging from 4.6 kg/0.25 cm^2^–5.5 kg/0.25 cm^2^ (21). The intercostal nerves which are part of the somatic nervous system innervate the sternum and the manubrium arises from the anterior rami of spinal nerves from a segment of T1–T11 (19, 21). It plays an important role in the contraction of intercostal muscles and also provides sensation to the skin. The pain impulses travel to the thoracic level of the spinal cord and synapse then continued to ascend as the second-order neuron.

## Peripheral Stimulus

### Periungual Pressure Technique Pathway

**Figure 4:**
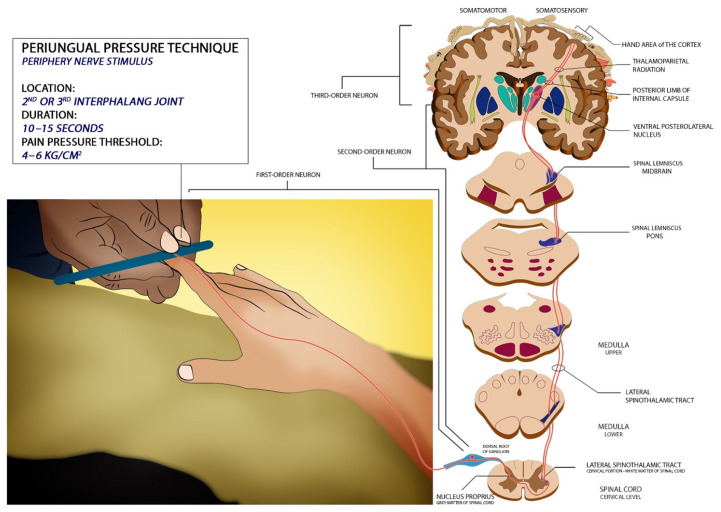
Illustration shows how to perform the periungual pressure technique and its prerequisite with related neurological pathways

The periungual pressure technique (Figure 4) involves applying pressure (400 kPa–600kPa ~ 4 kg/cm^2^–6 kg/cm^2^) (13) (with a pen to the lateral outer aspect of the second or third interphalangeal joint (applied with graduating intensity for 10 s–15 s) (3, 9). An accurate baseline needs to achieve, thus a necessity to repeat the protocol. The periungual or nail bed soft tissues are innervated by dorsal branches of paired digital nerves, running at the sides of the flexor tendon and with the dorsal neurovascular bundle (19). The typical ascending pathway for pain is being followed. Peripheral pain stimuli are not an effective way to test brain function (5, 9, 37).

### The Orders of the Neuron

#### First Order Neurons

The sensory receptors at the periphery or central are the starting point of 1st-order neurons of ascending pathway. As for the periphery, the digital nerves divide into three main branches just the distal to the distal interphalangeal joint. Each supplied to the nail bed, the tip of the digit, and the pulp (19, 22) as there are no specific innervation exists to show the type of innervation of their tips. Cutaneous innervation of the hand is mediated by the median nerve via a palmar digital cutaneous branch and a palmar cutaneous branch. Lateral three-and-a-half digits fingertips and the palmar surface of the hand supplied by the palmar digital cutaneous branch (19, 22). The index, middle, and ring fingertips are innervated by the palmar digital nerves meanwhile the thumb and little finger are innervated by the dorsal nerves. The lateral aspect of the palm is innervated via the palmar cutaneous branch which arises in the forearm and travels into the hand then it travels to the median nerve and joins the spinal cord via the brachial plexus (19, 22).

Both periungual and sternal rub technique pain stimuli transmit via will ascend at the spinal cord 1–2 vertebral levels above via the tract of Lissauer, and synapses at the tip of the dorsal horn at the grey matter of the spinal cord (11, 19). Thermal and mechanical nociceptors terminate in Rexed laminae I and V despite it is also contributing mainly to visceral organs (6, 12). Peripheral pain is transmitted predominantly via Aδ fibres to the dorsal (posterior) horn of the spinal cord via dorsal (posterior) spinal root ganglion (Antero Lateral System) (6, 8, 11, 19, 30). Most of the Aδ fibres end in the marginal zone (Rexed laminae I) (19). Glutamate is the neurotransmitter released here. Meanwhile, C-fibres polymodal nociceptors transmit their signals into the dorsal horn (6, 12, 19, 30, 37). For supra-orbital pressure and the jaw margin pressure technique, the pain stimuli will synapse at the sensory nucleus of the trigeminal nerve pars caudalis ipsilaterally at the lower medulla level. And then courses contralaterally to second-order neurons.

#### Second-Order Neurons

Pain or the stimuli information carried to the thalamus from the substantia gelatinosa via the second-order neurons. Periungual and sternal rub pressure technique pathways transmit pain via axons that immediately decussate and ascend along the lateral spinothalamic tract (STT) (pain and temperature) of the cervical and thoracic segments of the spinal cord to the lower part of the medulla (11, 12, 19). They are considered single pathways although they are functionally distinct. These ascending fibres are layered in the tract itself and actually run along with each other. These courses are cranially within the spinal cord. The spinal cord’s innermost medial fibres are from the highest part of the extremities, meanwhile the outermost contribution from the lowest such as from the sacral and also lower limbs (19). Finally, synapsing in the thalamus.

The supra-orbital and the jaw margin pressure technique transmit pain from the sensory nucleus pars caudalis of the lower part of the medulla via axons that immediately decussate and ascend along the ventral trigeminal tracts thru the sensory part of the trigeminal lemniscus. Finally synapsed at the thalamus for third order neuron.

Simultaneously, sensory information can be interpreted at a spinal level and the remaining sensory neurons can immediately synapse with motor neurons within the spinal cord, innervating a muscle and causing rapid, involuntary contraction and movement of the limb, thereby activating a reflex or withdrawal action by completion of the reflex arc (3). This explains the poor reliability of the peripheral stimulus over the central stimulus.

#### Third-order Neurons

Transmission of information to the somatosensory cortex is via the third-order neurons which carry the sensory signals from the thalamus to the ipsilateral primary sensory cortex of the brain (6, 11, 19). Sensory information ascends ipsilaterally from the ventral posterolateral nucleus of the thalamus via superior thalamic radiation (6, 11, 19). Then, it reaches the sensory cortex through the posterior limb of the internal capsule.

### Brainstem as Interchange Pain Highway

The brainstem is depicted as a neural highway. The reticular formation (RF) begins in the diencephalon (thalamus), extending caudally to the medulla oblongata, and projecting into superior cervical cord segments. It regulates consciousness by serving as one of the major integration and relay centre for brain function (11, 23). These complex net-like structures are one of the first brain structures that receive connections via spinoreticular tracts (ascending pain pathways in the spinal cord) at the medulla (11, 23). Originating from laminae V to VII, the spinoreticular fibres accompany STT thru the brainstem and terminate at all levels of the brainstem but are not exactly somatotopically arranged. Meanwhile, layers I, V and VI of the caudal spinal of trigeminal nucleus subnuclei have trigeminothalamic fibres which provide information for pain perception in the facial region (7). Impulse traffic is continued rostrally to the thalamus in a component of the ascending reticular activating system (ARAS) (23, 25). Briefly, the spinoreticular system has two interrelated functions which are to arouse the cerebral cortex, that is, to induce or maintain the waking state and report the nature of a stimulus to the limbic cortex of the anterior cingulate gyrus (25, 32). Supraspinal components of descending pain pathway mainly consists of rostral ventromedial medulla (RVM), the dorsolateral pontomesencephalic tegmentum and the periaqueductal grey (PAG) (10). In response to noxious stimuli, amygdala’s central nucleus also known as ‘nociceptive amygdala’ activate which associated with the emotional-affective component of pain and modulation (10). The emotional response may be pleasurable or aversive. Pain stimuli activate the RF and eventually contribute to the reactions of awake and alertness (11). These impulses that can be affected by pain are also simultaneously able to alter heart rate, arterial blood pressure, respiration, and other vital functions (11, 23). Meanwhile, the spinotectal tract is another tract that runs close to the spino-olivary tract, and synapses with the superior colliculus of the contralateral midbrain (23). This tract carries afferent information necessary for the spinovisual or ‘tectospinal’ reflexes which coordinate the movement of the head in response to painful stimuli which can be seen upon performing a GCS assessment. Some of these fibres also synapse in the PAG matter, where the tract also plays role in pain gating.

### Thalamus is the Sensory Input System for Pain Stimuli

**Figure 5:**
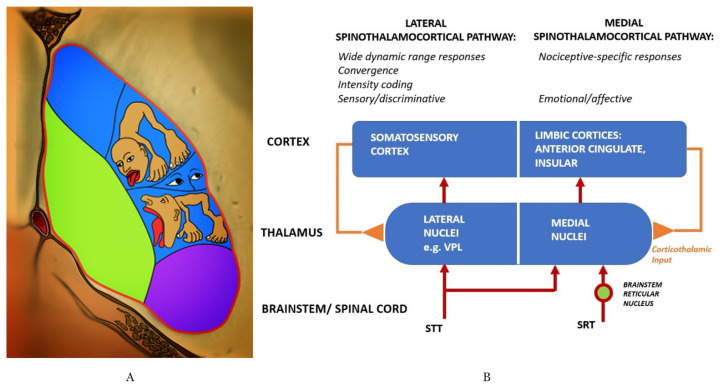
A: Illustration shows the somatotopic distribution of the thalamus. B: Schematic diagram showing a relation between neural structures with the thalamus in pain modulation (30)

One stop centre for the receipt, integration, transfer and modulatory role of nociceptive information is the small bean-shaped organ called the thalamus. Connections with various systems such as extrapyramidal motor, consciousness, visual, and limbic make it an important sensory input centre (11, 24). All information about pain regardless of the type, temporal pattern, intensity, cutaneous input and topographic localisation are encoded in the thalamus (Figure 5). The interaction with cortical and limbic structures gives sensory-discriminative and emotional dimensions of pain. The nociceptive inputs from the spinal cord to the thalamus are conveyed through the STT and in part through the spinoreticulothalamic tract (SRTT). The STT terminations projection are densely distributed throughout the medial and the lateral thalamus. The lateral thalamus consists of the ventroposterior lateral (VPL), VPM, ventroposterior inferior (VPI) and posterior (PO) nuclei. Meanwhile, intralaminar and submedius nuclei are in the medial aspect of thalamus (6, 11, 24, 25). The RF is also connected to the medial portion of the thalamus. Meanwhile, the VPL nucleus plays a major role in the sensory discrimination of pain (11). However, information regarding facial pain is spread over the trigeminal sensory nucleus complex (TSNC) and then ascends to the contralateral thalamus and cerebral cortex. TSNC consists of principal sensory nucleus and the spinal nucleus of trigeminal (7).

Thalamus has reciprocal connections (thalamocortical, corticothalamic) with the cerebral cortex (Figure 5B). Both intralaminar nuclei and reticular nuclei play a role in the cortical arousal response as they collectively receive fibres from several sources, motor and sensory, and project diffusely to the cerebral cortex (through other thalamic nuclei) (11, 24, 25, 26). The intralaminar nuclei connection with basal ganglia is involved in motor control mechanisms, and concurrently with input from ascending pain-mediating pathways, they are also involved in the awareness of painful sensory experiences. Certain afferents (thalamocortical fibres) specifically terminate within the functional columns of the cytoarchitecture cortex (25, 26). Thalamocortical radiations can be separated into four distinct radiations; anterior thalamic radiations (frontal lobe-limbic system), posterior thalamic radiations (parietal and occipital lobe), superior thalamic radiations (precentral and postcentral gyrus), and inferior thalamic radiations (insula, temporal lobe, and ventral portion of the frontal lobe) (24, 25). Once the information is processed, it will be relayed back from the cerebral cortex to the area of original activity in the thalamus via corticothalamic fibres (26). These corticothalamic projections, synapse with the thalamic reticular neurons and initiate the thalamic burst as pain response (24, 26, 27). Pain stimulation of the upper extremity activates bilateral insular cortex, thalamus, cerebellum, premotor areas and inferior parietal lobule however facial pain do activate bilateral cerebellum and the anterior insula but only activates ipsilateral ACC and contralateral thalamus (7). Awake and sleep were promoted via the hypothalamus-brainstem mutual inhibition circuits that suppress each other’s activity (28). Meanwhile, vigilance state such as wakefulness by tonic firing and sleep by burst firing are determined by the firing patterns of thalamocortical networks (28).

### Sensory—Motor Interconnection as Microprocessor Unit of the Cortex

D’Ostilio and Garraux (29) narrated higher-level areas of involvement in voluntary motor action from unconsciously perceived external stimuli. Thus evidence shows the sensory and motor cortex integration will be the microprocessor unit for all the information. Studies have shown that sensory-motor interconnectivity is as fast as 350 ms where preparatory motor activity even before brain became aware of the decision to act (29). Pain stimulus will reach the primary sensory cortex (Brodmann area 3, 2, 1) further the information will be processed in the secondary somatosensory cortex. Build-in interconnection of the cytoarchitecture of the motor-sensory cortex via direct and indirect tracts and also cortio-cortico connections relay sensory signals from the posterior parietal cortex and primary sensory cortex pertinent information such as somatosensation, proprioception and visuomotor transformations to the primary motor area (31, 37). The primary sensory cortex has a strong reciprocal connection with the primary motor cortex (Brodmann area 4), whereas the posterior parietal cortex has a weaker connection with the primary motor cortex. Sensory information will transmit via the premotor cortex before reaching the primary motor cortex (27, 31, 37). The white matter tract that is responsible for his connection is the superior longitudinal fasciculus. Hereby the information will exchange from impulses electrochemically. The six-layered Korbinian Brodmanns’ cytoarchitecture is subdivided into numerous areas based on regional differences (31). In terms of the level of cytoarchitecture, cortico-cortico connections between the sensory and motor cortex are found in a different layer of the cerebral cortex. Cytoarchitecture of the brain is the polymorph densely distributed of cells in cortical layers and sublayers which plays a role as the microprocessor. The fundus of the central sulcus is usually the margin between the primary motor and primary somatosensory areas (31). Pain stimulus input is relayed from the thalamus to layer 4 (cell-packed granular layer) of the primary sensory cortex and then transmit to layers 2 and 3 of the primary sensory cortex. Layer 2 and layer 3 of the primary sensory cortex will then send glutamatergic projections onto excitatory neurons in layer 2 and layer 3 of the primary motor cortex. The information is transferred to descending output Layer 5b (contains the giant Betz cells). Subsequently course to the pyramidal tract output (corticospinal tract (~30%); Premotor cortex and the supplementary motor area (~30%), the somatosensory cortex (~30%), and the posterior parietal cortex (~10%) which causes overall motor movement (24). Moreover, reciprocal connections between the sensory and motor cortex are seen in layer 5a.

### Clinical Significance of Pain Stimuli in GCS Status

Clinicians always struggle or sometimes impossible to prove a patient is lacking awareness compared to awareness of an unresponsive patient (1). GCS interpretation of each component can be divided into cortical, subcortical, brainstem, and cervical-medullary junctions (32, 35). Awareness refers to the phenomenal perception of self and surroundings. Wakefulness describes the state of arousal or potential to experience awareness (23, 32). Amygdala, prefrontal cortex, cingulate cortex, basal ganglia and cortico-limbic reverberating loops have an interconnection that closely relates to human consciousness or GCS clinically (10).

#### Pain in Eye Response

A conscious person can open an eye spontaneously without any external stimulus indicating that arousal mechanisms in the brainstem are intact (E4) (35). It is associated with consciousness, which comprises two main components: awareness and wakefulness (16, 23). The key mediator of wakefulness is based on integrity of a pathway that is adjacent to the pontine RAS (38). In the eye component of GCS, opening an eye to pain (E2) via the painful stimulus is given at either the nail bed with a pen/pencil or pinching the trapezius muscle or rubbing the sternum (15, 32, 33, 35, 37). The stimulus is sustained for 10 s (if required) before concluding that there is no response to pain. Proprioceptive fibre from trigeminal mesencephalic is supplied for the eyelid opening via mechanoreceptors in the supratarsal Müller muscle (34).

#### Pain in Verbal Response

Two pathways were responsible for a verbal response. First, the limbic pathway authorises commands from the cerebral cortex (ACC) thru RF to the periphery motor via the PAG matter. This gives the initiation and intensity of vocalisation. The motor cortical pathway plots the final motor command from the motor cortex to the RF for the production of learned vocalisations. Later, integrate with the other two feedback loops involving subcortical structures that pre-process the motor commands. A loop via the pons, cerebellum and thalamus, and another through the putamen, pallidum and thalamus. Together, the components of the motor cortical pathway control the specific pattern of vocalisation (36). Thus, verbal assessment gives its complete value V5. V4 will give its value of impaired cortical subcortical level, meanwhile the V3 will include deep structure involvement such as limbic pathway and other deep structure up to the level above pontine. Voice production requires organised motoneurons pathways that orchestrate muscles of respiration, vocalisation, and articulation and converge onto the RF of the pons and medulla oblongata (36). The role of pain stimulus in verbal is crucial. V2 or incomprehensible sounds, the patient moans, or groans, most often seen for a painful stimulus. Thus it gives an idea to clinicians regarding the localisation of defects (15, 33, 35).

#### Pain in Motor Response

Basal ganglia circuits play an important role in the control of movement planning and execution, notably in initiating, inhibiting, and switching behaviours via interaction between motor, cognitive and limbic systems (29). Localising to a painful stimulus upon motor assessment or M5 is the response when the hand reaches the site of stimuli such as the sternum or trapezius (15, 37). When the supra-orbital pressure technique is applied, the patient’s hand even goes above the level of the clavicle but does not necessarily reach the site of the stimulus. A common misconception about the GCS is the equation of abnormal flexion and extensor response to decorticate and decerebrate rigidity respectively.

**Figure 6:**
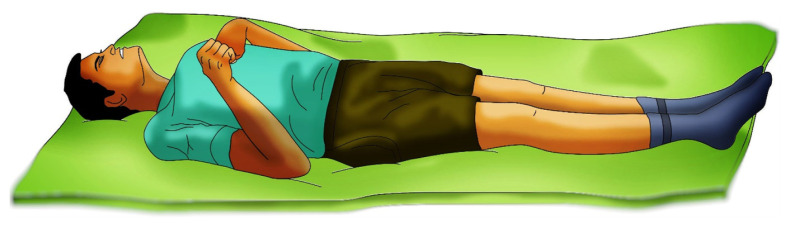
Decorticate posturing upon pain stimuli explains the insult above the midbrain and thus may have a better prognosis

In head injury, the severity correlates with the GCS score irrespective of the site. A poor motor response can be a result of severe cortical or hemispheric lesions. M3 and M4 (normal/abnormal flexion) the importance to document these responses are if the patient demonstrates any degree of flexion (32, 33, 35). The patients who show any degree of flexion response on a persistent basis do well overall than those who have extensor posturing (Figure 6). A flexion response in the lower limbs also can be observed (25, 30).

**Figure 7:**
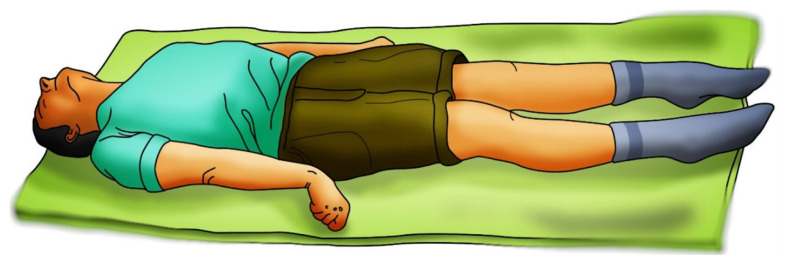
Decerebrate posturing explains the insult below the midbrain and thus may have a poor prognosis

The patient who is not localising to a painful stimulus is more likely to be in the severe than moderate head injury group. A range of movement is possible in a patient who is not localising to a painful stimulus. It can vary from a ‘normal flexion’ which is rapid withdrawal and abduction of the shoulder with external rotation. In ‘abnormal flexion’ there is adduction and internal rotation of the shoulder (classical decorticate posture (Figure 7) (32, 33, 35, 37).

Between these two extremes of movements, there may be varied patterns and also both types of movements may be seen at the same time in a patient. One simple and practical way to sort out the two responses is by observing the position of the forearm. Score as M3 if the forearm is in pronation position. Then as M4 if the forearm is in supination without localising to the painful stimulus (32, 33, 35). In the situation of a difference in the motor score, it is best to manage as per the lower score.

Apart from that, at the diencephalon level especially thalamus as the regulator of the global brain state, and its interactions with deep cortical layers (thalamocortical pathway) which gives awareness, attention and cognition plays a crucial role in assessment of the GCS. Thus an early changes in consciousness level were able to observed. These changes can gives a clinical person a clue regarding consciousness level of the patient and noxious stimulus warranted at this level and below. Structurally, central lateral thalamus receives input from the ARAS yet another location plays and critical role in pain localisation as they extended from the diencephalon till the medullary level. The damage to the ganglia in peripheral system may gives a false-positive of the GCS level so it is necessary to use both central and peripheral technique for GCS assessment. The more detailed has been showed at the Table 1.

## Conclusion

Supraorbital Notch and Jaw Pressure mainly use the fifth cranial nerve sensory pathway. Meanwhile, the sternal rub and nail bed stimulus used a classical root ascending spinothalamic pathway. Pain is an essential key or tool that guides GCS status assessment, especially in an unconscious person. It gives a significant clue for clinicians whereabouts the neurological defect status of the patient. Understanding the pain pathway and technique to elicit noxious stimuli will help clinicians to accurately assess GCS and possible localisation of the affected site in human nervous system.

## Figures and Tables

**Table 1:** Summary of the localisation, best GCS and its clinical explanation

Level	Affected location		Best GCS level			Grady Coma Scale	Clinical explanation

			E	V	M		
A. Central nervous system							

Cortex	Somatosensory		3	5	5	Grade I: Confused	Not fully conscious but aware the noxious stimulus Spinoparabrachial-amygdala pathway intact All pain related tracts intact
	Motor						
Corona radiata	Thalamocortical pathway		3	4	5	Grade II: Disorientated	Spinoparabrachial-amygdala pathway intact ARAS Intact with brainstem Confused or single word speech
Internal capsule	Posterior limb		3	3	4		
Thalamus	VPM/VPL	ARAS network disrupted	2	3	4	Grade III: Lethargic/Obtunded	Spinoparabrachial-amygdala pathway intact Thalamocortical pathway impaired (6) Spino reticular thalamic tract impaired (6) Spino-hypothalamic tract impaired
Midbrain	Upper		2	3	3	Grade IV: Stuporous	Spinomesencephalic impaired Pupil Midposition (1 mm–3 mm) (INO if MLF affected) Cheyne’s stoke breathing at the level of diencephalon DECORTICATE contralateral to the affected site
	Lower		1	3	2		Trigeminal mesencephalic nucleus affected Trigeminothalamic tract impaired (7) Pupils 3 mm–5 mm fixed and dilated Unable to open eyelid (34) DECORTICATE → DECEREBRATE
PONS	Upper		1	2	2		Spinoparabrachial-amygdala pathway impaired (6) Pupils pinpoint due to uninhibited sympathetic activity Pneumotaxic breathing Usually corneal reflex impaired (38) Spino reticular thalamic impaired Only sound verbally
	Lower		1	2	2		Pupil midposition (3 mm–5 mm) Cluster breathing
Medulla	Upper		1	1	1	Grade V: Comatose	PAG-rostral ventromedial medulla (PAG-RVM) system impaired Lateral and caudal dorsal reticular nucleus (DRt) and ventrolateral medulla (VLM) impaired Pupils fixed and dilated (7 mm–10 mm) Ataxic or Biot’s breathing Spino reticular thalamic impaired (6)
	Middle						
	Lower						
Spinal cord (post-ganglionic)	Cervical						Central stimulus are recommended for GCS assessment
	Thoracic						Sternal rub technique not recommended for GCS assessment

B. Peripheral nervous system**							

Ganglia	Cervical		3	5	5	Grade I or II	Spinocervicothalamic pathway impaired (6) Peripheral stimulation UNABLE to applicable in view it may give false positive on the GCS level
	Thoracic						
	Lumbar						ALL stimulus techniques APPLICABLE
	Trigeminal						Supraorbital and jaw margin technique NOT APPLICABLE
	Muscle		4	5	6		All techniques applicable provided the patient is full consciousness

Note:

** Only applicable if ARAS intact
